# β-Glucan-Induced Trained Immunity in Dogs

**DOI:** 10.3389/fimmu.2020.566893

**Published:** 2020-10-09

**Authors:** Simon Paris, Ludivine Chapat, Marion Pasin, Manon Lambiel, Thomas E. Sharrock, Rishabh Shukla, Cecile Sigoillot-Claude, Jeanne-Marie Bonnet, Hervé Poulet, Ludovic Freyburger, Karelle De Luca

**Affiliations:** ^1^Boehringer Ingelheim Animal Health, R&D, Lyon, France; ^2^Université de Lyon, APCSe, Pulmonary and Cardiovascular Agression in Sepsis, VetAgro Sup-Campus Vétérinaire de Lyon, Marcy l'Étoile, France; ^3^Département Biologie, Faculté des Sciences et Techniques, Université Claude Bernard Lyon 1, Villeurbanne, France; ^4^Lifebit Biotech Ltd, London, United Kingdom

**Keywords:** trained immunity, monocytes/macrophages, epigenetic, immuno-metabolism, veterinary immunology, comparative immunology, canine (dog)

## Abstract

Several observations in the world of comparative immunology in plants, insects, fish and eventually mammals lead to the discovery of trained immunity in the early 2010's. The first demonstrations provided evidence that innate immune cells were capable of developing memory after a first encounter with some pathogens. Trained immunity in mammals was initially described in monocytes with the Bacille Calmette-Guerin vaccine (BCG) or prototypical agonists like β-glucans. This phenomenon relies on epigenetic and metabolic modifications leading to an enhanced secretion of inflammatory cytokines when the host encounters homologous or heterologous pathogens. The objective of our research was to investigate the trained immunity, well-described in mouse and human, in other species of veterinary importance. For this purpose, we adapted an *in vitro* model of trained innate immunity in dogs. Blood enriched monocytes were stimulated with β-glucans and we confirmed that it induced an increased production of pro-inflammatory and anti-microbial compounds in response to bacterial stimuli. These results constitute the first demonstration of trained immunity in dogs and confirm its signatures in other mammalian species, with an implication of cellular mechanisms similar to those described in mice and humans regarding cellular epigenetics and metabolic regulations.

## Introduction

Innate immunity was originally described by its immediate and short-lived response, and its capacity to react to a broad spectrum of pathogens without specific recognition as well as its lack of memory. However, the discovery of Toll-like receptors in the *Drosophila* species showed that innate immunity is able to display pathogen-class specificity through the pattern recognition receptors (1, 2). Research in plants then showed that even though they exhibit an incomplete adaptive immune response, they still possess a form of immune memory linked to epigenetic reprogramming of immunocytes (3). Since then, evidence in favor of a form of memory, named trained immunity has been found in a very diverse set of organisms, from plants and insects (4) to fish (5) and eventually mammals, showing its undisputable evolutionary role (6). The first demonstrations in mouse models showed that the innate immune response can remember a first encounter with some pathogens leading to an enhanced secretion of inflammatory cytokine (i.e., TNF-α, IL-6, IL-1β) when the host encounters homologous or heterologous pathogens (7).

Trained immunity acts in a T- and B-cell independent manner and involves macrophages, dendritic cells and natural killer cells (8–10). It was recently demonstrated that the training of monocytes can be achieved by two main signaling pathways mediated through pattern recognition receptors (PRRs) (11), NOD-like receptors (NLRs), i.e., NOD2 (12), and C-type lectin receptors (CLRs) (9). Amongst the latter, Dectin-1 binds polymers of glucose as well as a specific binding site on CD11b, constituting with CD18, the complement receptor 3 (CR3) (13, 14). Several pathogen associated molecular patterns (PAMPs) can induce trained immunity. Different prototypical agonists, like muramyl-dipeptide (MDP) for NOD2, β-glucans to Dectin-1, or BCG vaccine, have been described to act as training compounds (15).

Current knowledge suggests that trained immunity is orchestrated and regulated by epigenetic reprogramming and associated metabolic changes (16, 17). These changes result in sustained modifications of gene expression and cellular processes. The causal relationship between epigenetics and metabolism still remains to be proven but is thought to occur in positive feedback loops (18). Indeed, specific histone marks have been described to enhance transcription of several gene clusters, notably some implicated in the regulation of cell metabolism. The two main types of post-translational histone modification described in trained immunity are acetylation and methylation (19). These marks can act as activator or repressor toward transcription depending on the amino acid being modified. Tri-methylation of the fourth lysine residue (H3K4me3) and acetylation of the 27th lysine residue (H3K27Ac) of the histone H3 are defined as active enhancer marks resulting in transcription activation. These two marks are well-described in the trained phenotype of macrophages (17).

The maintenance of the trained phenotype is also enabled by a metabolic switch (20). This metabolic change, known as the Warburg effect, arises in highly transcriptionally active or proliferative conditions. Basal cell metabolism mostly relies on oxidative phosphorylation. This mechanism displays a tendency to increase glycolysis while decreasing oxidative phosphorylation. This higher glycolytic flux is directed toward lactate production. This process, called aerobic glycolysis, was demonstrated in trained innate cells and is therefore used as a validation of immune training (21). Aerobic glycolysis and the use of alternate source of energy, such as glutaminolysis, will favor the accumulation of certain metabolites of the tricarboxylic acid (TCA) cycle (16). Those metabolites can be used as cofactors of histone-modifying enzymes, reinforcing the activity of these enzymes and thus histone modification (18). Overall, these modifications at the metabolic and epigenetic level cause the increase of the pro-inflammatory and anti-microbial responses of innate immune cells (22).

The clinical implications of trained immunity for vaccine development as well as adjuvants is a new area or research (23). However, limited data are available regarding the existence of trained immunity mechanisms in small or large mammals, and in companion or production animals (24, 25). Targeting trained immunity sets two main goals, firstly enhancing the general resistance of animals in critical life periods, such as young age, stress periods, or in elderly population; and secondly increasing vaccine intake by new mechanisms of adjuvantation (26).

In the proposed work, we focused on dogs and implemented an *in vitro* model of immune training adapted from the literature (27). Different PAMPs were screened to check their training or tolerogenic abilities based on the increased or decreased cytokine secretion, a hallmark of trained immunity (TNF-α, IL-6, IL-1β) (23). After identifying the most effective compounds, we analyzed the features of trained immunity. Combining immunology, epigenetic and metabolic features, we showed the ability of canine macrophages to undergo the modifications characterized in trained immunity models, validating the concept of trained innate immunity in additional species.

## Materials and Methods

### Animals and Biological Sampling

The Boehringer Ingelheim Ethical Committee (registered under n° 13 at the French Ministère de l'Education Nationale, de l'Enseignement Supérieur de la Recherche) has reviewed all animal experiments conducted for this study before conducting the study. Approval confirms that all experiments conform to the relevant regulatory standards defined by the European and French Laws (directive EU2010/63 and Decret n°2013-118). Peripheral blood samples from Beagle puppies of 4 to 10 weeks of age and of male and female were collected into heparinized tubes. Dogs, provided by a commercial supplier, were maintained in conventional conditions on a commercial diet.

### Isolation and Culture of Blood-Derived Monocytes

Canine peripheral blood mononuclear cells (PBMCs) were extracted from whole blood of healthy animals by density-gradient centrifugation over human Pancoll (density 1.077 g/ml, PAN Biotech, D. Dutscher, Issy-les-Moulineaux, France) after centrifugation for 30 min at 1,100 g at room temperature (RT). PBMCs were counted using a Pentra DF Nexus cell counter (HORIBA ABX SAS Medical, Montpellier, France) and re-suspended to the required concentration in RPMI 1640 (Life Technologies, Villebon sur Yvette, France) supplemented with 10% irradiated fetal bovine serum, 1% penicillin-streptomycin (Life Technologies, Villebon sur Yvette, France) and 0.01% β-mercaptoethanol (Sigma-Aldrich, Saint-Quentin-Fallavier, France). After extraction from whole blood, cells were seeded at a concentration of 2 × 10^6^cells/well (6.7 × 10^6^ cells/cm^2^) in 96-well culture microplates (CORNING, 353072, Falcon 96-well Clear Flat Bottom TC-treated Culture Microplate). Cells were incubated at 37 ± 2°C in a humidified incubator with 5% CO_2_ for 2 h. Adherent monocytes were selected by washing out non-adherent cells with warm PBS without Ca^2+^ or Mg^2+^.

### Immuno-Stimulation and Immune Training Experiments

#### Immune Training Experiments

Protocols were established based on Quintin et al. (9). Briefly, after extraction and seeding, monocytes were stimulated/trained with several stimulatory compounds (Table 1) for 24 h or left untreated (negative control—NA) hereinafter called “priming” step. Supernatants were then collected and stored at −80°C for further analysis and cells were wash gently 2 times with pre-warmed culture medium (RPMI 1640 (Life Technologies, Villebon sur Yvette, France) supplemented with 10% irradiated bovine calf serum, 1% penicillin-streptomycin [Life Technologies, Villebon sur Yvette, France) and 0.01% β-mercaptoethanol (Sigma-Aldrich, Saint-Quentin-Fallavier, France)]. After the priming step, cells rested for 6 days incubated at 37 ± 2°C in a humidified incubator with 5% CO_2_, with fresh culture medium replenishment after 3 days in culture. At the end of the resting period supernatants were harvested and stored at −80°C for further analysis. Cells were then stimulated for another 24 h period with several stimulatory compounds listed below to mimic an immune challenge called the “challenge” step. Finally, cells and supernatants were harvested for characterization of different immunological parameters such as cytokines secretion, cell surface markers and reactive oxygen species measurement, as well as metabolites quantification. At the 3 different time points described earlier—priming step; resting step and immune challenge step—cells were subjected to any analysis for better describing the trained innate immunity in canine monocytes.

**Table 1:** Stimulatory compounds.

**Compound**	**Species**	**Description**	**Receptor**	**Manufacturer**	**Catalog#**	**Concentration**
Pam3CSK4	Synthetic tripalmitoylated lipopeptide	/	TLR1/2	Invivogen	tlrl-pms	10 μg/mL
LPS	*Escherichia coli*	Strain O55:B5	TLR4	SigmaAldrich	L2637	10 ng/mL
LPS	Pseudomonas aeruginosa	Strain 10	TLR4	SigmaAldrich	L8643	10 ng/mL
Zymosan	Saccharomyces cerevisiae	Branched polymer of β(1-3)-D-glucose with β(1-6)-linkages	TLR2 Dectin-1	Invivogen	tlrl-zyn	10 μg/mL
β-glucan from Euglenids (Paramylon)	Euglena gracilis	Linear polymer of β(1,3)-D-glucose	Dectin-1 (lack of TLR agonistic activity)	SigmaAldrich	89862	10 μg/mL
Whole Glucan Particule (WGP)	Saccharomyces cerevisiae	Branched polymer of β(1-3)-D-glucose with β(1-6)-linkages	TLR2 Dectin-1	Invivogen	tlrl-wgp	10 μg/mL
Laminarin	Laminaria digitata	Branched polymer of β(1-3)-D-glucose with β(1-6)-linkages	Agonist or antagonist of Dectin-1 depending on the laminarin preparation	Invivogen	tlrl-lam	100 μg/mL
Curdlan	Alcaligenes faecalis	Linear polymer of β(1,3)-D-glucose	Dectin-1 (lack of TLR agonistic activity)	Elicityl	GLU511	10 μg/mL
Dextran	Not documented	Branched polymer of α(1,6)-D-glucose with α(1,3)-linkages	DC-SIGN	SigmaAldrich	00268	10 μg/mL

#### Stimulatory Compounds

A total of 9 compounds were used to stimulate the cells, either as first stimulation inducing the training or as secondary stimulation allowing to measure the effects of the training on canine macrophages. These compounds are further described in Table 1.

#### Inhibition of Priming Steps

For inhibition of histone methylation, monocytes were pre-incubated for 1 h with 5'-Deoxy-5'-(methylthio) adenosine at 3 mM ([MTA], D5011, Sigma-Aldrich, Saint-Quentin-Fallavier, France) before priming with the desired compounds. For inhibition of glycolysis, monocytes were pre-incubated with 2-Deoxy-D-Glucose at 15 mM ([2-DG], D6134, Sigma-Aldrich, Saint-Quentin-Fallavier, France). No washes were performed after the pre-incubation, the usual training protocol followed with a 24 h period of priming. The viability of the cells was analyzed post pre-treatment and priming, to characterize the best working concentration of the inhibitors with a threshold settled at 80% of viable cells analyzed by flow cytometry. Inhibition was finally estimated by the impact on the final secretion of cytokines after challenge.

### Cytokines Secretion Measurement

#### Quantification of Cytokines by Enzyme-Linked Immunosorbent Assays (ELISA)

Supernatant levels of IL-6, TNFα, IL-1β were detected using canine DuoSet ELISA kits (R&D Systems, Minneapolis, MN; catalog #DY1609; #DY1507 and #DY3747, respectively) according to the manufacturer's instructions. The optical densities of the peroxidase product were measured by spectrophotometry using a Synergy 2 microplate reader (Biotek, Winooski, VT) at a wavelength of 450 nm.

#### Quantification of Cytokines by ProcartaPlex Assays

ProcartaPlex assays use Luminex™ xMAP technology for the multi-analyte detection of secreted proteins. The concentration of multiple canine cytokines (IL-2, IL-6, IL-8, IL-10, TNFα, IL-12p40, IFN-γ, MCP-1, SCF, β-NGF, VEGFα) released into the cell-culture supernatant was measured using a LUMINEX kit (Affymetrix ProcartaPlex Canine 11-Plex, Ebioscience SAS, Paris, France) according to the manufacturer's recommendations. Sample fluorescence was read on a Bio-Rad Bioplex 200 System and analyzed using Bioplex Manager 6.1 software (Bio-Rad, Hercules, CA).

### Flow Cytometry

#### Instruments and Analyses

Cells were analyzed using a fluorescence-activated flow cytometer (BD FACSVerse™, BD Biosciences, San Jose, CA, USA) calibrated with BD FACSuite™ CS&T Research Beads; FC Bead 4c, FC Bead 4c+ and FC Bead Violet Research Kits (respective references 650621, 650625, 650626, 650627, BD Biosciences, San Jose, CA, USA). The distribution of doublets was assessed using a side scatter height (SSC-H) vs. side scatter area (SSC-A) density plot followed by a forward scatter height (FSC-H) vs. forward scatter area (FSC-A) plot. Debris and dead cells were assessed both based on forward and side scatter and with viability dyes LIVE/DEAD™ Fixable Dead Cell Stain Kits (Far Red for 633 or 635 nm excitation, L10120 or Green for 488 nm excitation, L23101; Thermo Fisher Scientific Inc., Waltham, MA, USA) before proceeding with the analysis. Acquired events were recorded using BD FACSuite software and analyzed using FlowJo v10.0.7, LLC software. GraphPad Prism software was used for statistical analysis of compiled flow cytometry data.

#### Quantification of Reactive Oxygen Species (ROS)

Quantification of Reactive Oxygen Species [ROS] namely hydrogen peroxide, peroxynitrite and hydroxyl radicals was achieved using the ROS-ID® Total ROS detection kit (ENZ-51011, ENZO Life Sciences, Inc.). All experiments were performed following the manufacturer's recommendations. ROS intracellular levels were examined using a 488-nm wavelength excitation on FACS VERSE. Acquired events were recorded using BD FACSuite software and analyzed using FlowJo v10.0.7, LLC software.

### Analysis of Phagocytosis Using Real-Time Incucyte® Live Cell Analysis System

For pHrodo assays, cells were cultured in 96-well plates and added to a single tube of lyophilized pHrodo Red *E. coli* BioParticles (Thermo Fisher Scientific). The BioParticles were vortexed and then incubated for 30 min in a 37°C water bath. BioParticles were centrifuged at 2,000 rpm for 2 min and washed twice with PBS (pH 7.4, without Ca and Mg), and resuspended in 2 ml RPMI-1640 with 10% FBS. BioParticles were added to the plate immediately before being placed into IncuCyte high-throughput live cell microscope system (Essen BioScience, Ann Arbor, MI, USA) for detection of RFP expression. Cells in a 96-well plate were imaged through a 10× objective lens at 20 min intervals for 10 h using the IncuCyte S3 HD live cell imaging system (Essen BioScience). The system was located in a 37°C/5% CO_2_ cell culture incubator to maintain proper incubation conditions. Analysis was performed using the default red fluorescence processing definition within the IncuCyte S3 2019B software. The number of red cells and confluence of measured red fluorescence were translated from the images and exported using the Excel format.

### Metabolomics Analysis: Metabolon, Inc. (USA, Morrisville, NC 27560)

#### Sample Preparation

Supernatants and cells were harvested throughout the immune training protocol and stored at −80°C before preparation for the analysis. Samples were prepared using the automated MicroLab STAR® system from Hamilton Company. Several recovery standards were added prior to the first step in the extraction process for QC purposes. Samples were extracted with methanol under vigorous shaking for 2 min (Glen Mills GenoGrinder 2000) to precipitate protein and dissociate small molecules bound to protein or trapped in the precipitated protein matrix, followed by centrifugation to recover chemically diverse metabolites. The resulting extract was divided into five fractions: two for analysis by two separate reverse phase (RP)/UPLC-MS/MS methods using positive ion mode electrospray ionization (ESI), one for analysis by RP/UPLC-MS/MS using negative ion mode ESI, one for analysis by HILIC/UPLC-MS/MS using negative ion mode ESI, and one reserved for backup. Samples were placed briefly on a TurboVap® (Zymark) to remove the organic solvent. The sample extracts were stored overnight under nitrogen before preparation for analysis.

#### Ultrahigh Performance Liquid Chromatography-Tandem Mass Spectroscopy (UPLC-MS/MS)

All methods utilized a Waters ACQUITY ultra-performance liquid chromatography (UPLC) and a Thermo Scientific Q-Exactive high resolution/accurate mass spectrometer interfaced with a heated electrospray ionization (HESI-II) source and Orbitrap mass analyzer operated at 35,000 mass resolution. The sample extract was dried, then reconstituted in solvents compatible to each of the four methods. Each reconstitution solvent contained a series of standards at fixed concentrations to ensure injection and chromatographic consistency. One aliquot was analyzed using acidic positive ion conditions, chromatographically optimized for more hydrophilic compounds. In this method, the extract was gradient-eluted from a C18 column (Waters UPLC BEH C18-2.1 × 100 mm, 1.7 μm) using water and methanol, containing 0.05% perfluoropentanoic acid (PFPA) and 0.1% formic acid (FA). A second aliquot was also analyzed using acidic positive ion conditions, but was chromatographically optimized for more hydrophobic compounds. In this method, the extract was gradient eluted from the aforementioned C18 column using methanol, acetonitrile, water, 0.05% PFPA and 0.01% FA, and was operated at an overall higher organic content. A third aliquot is analyzed using basic negative ion optimized conditions using a separate dedicated C18 column. The basic extracts were gradient-eluted from the column using methanol and water, however with 6.5 mM Ammonium Bicarbonate at pH 8. The fourth aliquot was analyzed via negative ionization following elution from a HILIC column (Waters UPLC BEH Amide 2.1 × 150 mm, 1.7 μm) using a gradient consisting of water and acetonitrile with 10 mM Ammonium Formate, pH 10.8. The MS analysis alternates between MS and data-dependent MSn scans using dynamic exclusion. The scan range varied slightly between methods, but covered ~70–1,000 m/z. Raw data files were archived and extracted as described below.

### Statistical Analyses

Statistical analyses and graphical representation were performed using the softwares Minitab 19 Statistical Software and GraphPad Prism 8.

### Dectin-1 Structural comparisons

Murine Dectin-1 structure co-crystallized with a β-glucan published in the Protein Data Bank (PDB) was used as template to model the different structures of the Dectin-1 variants. Modeling of human and dog Dectin-1 structures was conducted upon these templates with Swiss-model automated protein structure homology-modeling server (28). Models were visualized using Maestro 11.8 software and sur-impressed to localize the amino-acids involved in β-glucan interaction and compare their spatial arrangements.

## Results

### β-glucans Are Able to Induce Trained Immunity in Canine Macrophages

**Figure 1:**
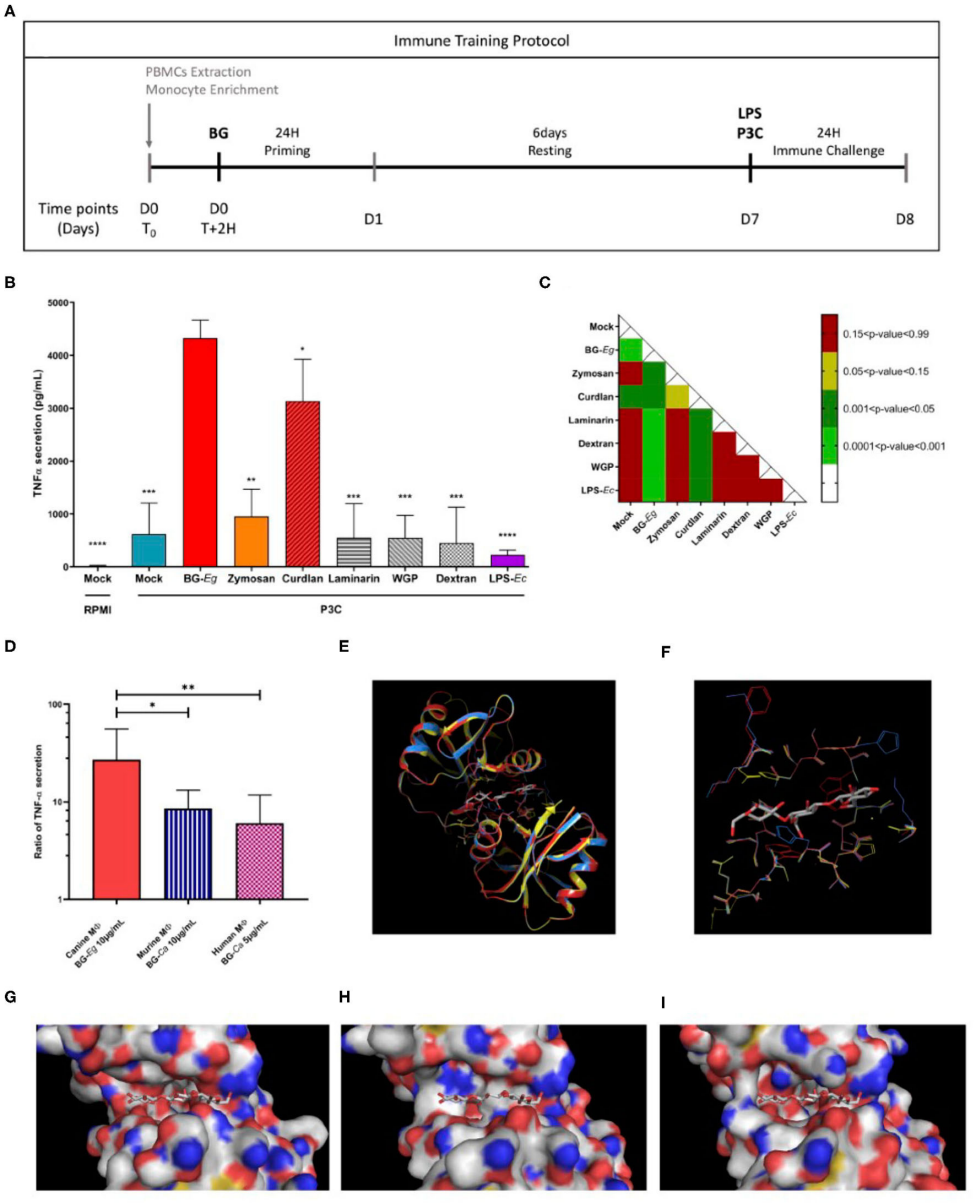
Comparison of immune training of macrophages using different stimulating compounds. **(A)** Experimental design of immune training model *in vitro*. **(B)** TNF-α secretion after a full immune training protocol at D8 (Means ± SD). Statistic comparison to BG-Eg primed macrophages. **(C)** *p*-values of mixed-effects analysis of one way ANOVA between TNF-α secretions. **(D)** Ratio of TNF-α secretion between control condition (Mock/LPS) and primed macrophages in different species. 3-D representation of murine Dectin-1 -2CL8 (yellow) co-crystallized with a β-glucan- superimposed with modeling of human (red) and dog (blue) corresponding proteins. **(E)** Globlal overview of the proteins. **(F–I)** Focus on amino-acids at 4 angstrom distance from the crystallized β-glucan. Dectin-1 structures from the three species are represented superimposed **(F)** or individually: murine in yellow **(G)**, human in red **(H)** and dog in blue **(I)**. ANOVA tests *p*-values * < 0.05, ** < 0.01, *** < 0.001, **** < 0.0001.

To assess the ability of canine macrophages to be trained, the experimental model of immune training described below was adapted from Quintin et al. (9) (Figure 1A). Macrophages were stimulated using a panel of PAMPs targeting Dectin-1 and/or TLR2 or TLR4 PAMPs for 24 h (D1). After a resting period of 6 days without any stimulating agents and an immune challenge of 24 h (D8) using either LPS from *E. coli* or P3C (Pam3CysSerLys4) was performed and TNF-α secretion was assessed at the end of the protocol as shown in Figure 1B. Macrophages stimulated with P3C in absence of priming displayed a heightened secretion of TNF-α compared to the basal level of secretion in mock/RPMI condition.

Canine macrophages primed with β-glucan from *E. gracilis* (hereafter BG-*Eg*) displayed the highest production of TNF-α, statistically different from every other compounds. The other compounds could be divided into 3 groups. A first group in which priming with LPS from *E. coli* (LPS-*Ec*) induced a lower TNF-α secretion than the one observed for unprimed cells (*p*-value 0.071), corresponding to some tolerogenic effects. A second group primed with either Laminarin, Dextran, or WGP displayed a TNF-α secretion in the same range as the control condition Mock/P3C, significantly lower than the one observed for BG-*Eg* priming. Finally, priming with Zymosan or Curdlan showed an intermediate increase of TNF-α secretion after P3C re-stimulation, significantly higher than the control condition Mock/P3C, but inferior to the one observed for BG-*Eg* priming (*p* < 0.01 and <0.05, respectively). All challenged conditions were compared to one another using a one-way ANOVA test and *p*-values are presented in the matrix in Figure 1C. Priming with β-glucan from *E. gracilis* (BG-*Eg*) and Curdlan are the only significant groups when compared to control condition (Mock) and showing statistical significance to all other compounds as well. The β-glucan from *E. gracilis* showed the highest increase, statistically significant when compared to Curdlan (*p* < 0.05). Thus, the compound selected as the best training inducer was the β-glucan from *E. gracilis;* then used as the reference compound for the rest of the results presented in this work.

Using raw data kindly provided by Quintin et al. (9) and Ian Bekkering et al. (29), we calculated the ratio of TNF-α secretion from primed conditions (β-glucan/LPS) vs. control conditions (Mock/LPS). β-1,3-(D)-glucan from *Candida albicans* and β-1,3-(D)-glucan from *Saccharomyces cerevisiae* were, respectively, used to prime murine (9) and human macrophages (29). In Figure 1D, we compared the TNF-α ratios to the ones obtained in our experimental set up with canine macrophages stimulation with β-1,3-(D)-glucan from *Euglena gracilis* in our study. Similar increased ratios of TNF-α secretion were found in-between species, varying from 10-fold increase for murine and human macrophages to almost 30-fold increase for canine macrophages.

### Impact of Structures of β-Glucan on Trained Immunity

Human and dog recognition site of Dectin-1 were modeled using the murine recognition site of Dectin-1 structure co-crystallized with Laminatriose, a natural β-glucan (PDB reference 2CL8). The modeled Dectin-1 sequences of the two species were compatible with the murine structure used as a template. Their highly similar structures shown in the Figure 1E, underline the evolutionary conservation of Dectin-1 and thus its biological role. However, if we are looking more specifically in the groove involved in the Dectin-1/β-glucan interaction, some major differences in the amino-acid composition could be noted (Figures 1F–I). For dogs in particular, the length of amino-acids present at the groove entry is longer and induces an increase of the steric hindrance. For example, the small and neutral amino-acid Glycine G152 on mice and human Dectin-1 is occupied by a histidine in dogs, a larger and polar amino-acid. Moreover, changes in the amino-acid composition induce some 3-D constraints and impact the amino-acid residues orientation. In canine Dectin-1, the Lysine K243 presents more sterical constraints than the same residue from the human one and this organization reduces the accessibility of the β-glucan in the Dectin-1 groove. These predictions can be put in perspective with the results of Figure 1D, in which we compare TNF-α secretion by macrophages from different species that underwent a full immune training. Following the same experimental procedures, the enhanced TNF-α secretion is globally conserved though it varies in terms of range (10-fold increase to 30-fold increase) compared to their control counterparts.

### β-glucan From *E. gracilis* Induces a Specific Cytokine Signature Associated With Trained Immunity

After deciphering the fittest model and candidate, we aimed to show the specific cytokine signature of trained immunity as well as its kinetics (27). We monitored along the whole protocol the cytokine secretion, namely TNF-α, IL-6, and IL-1β, markers of trained immunity. For each condition and time point of the experimental design, cells were harvested and counted by flow cytometry. The results were later used to index to cytokine secretion on the number of cells per wells.

**Figure 2:**
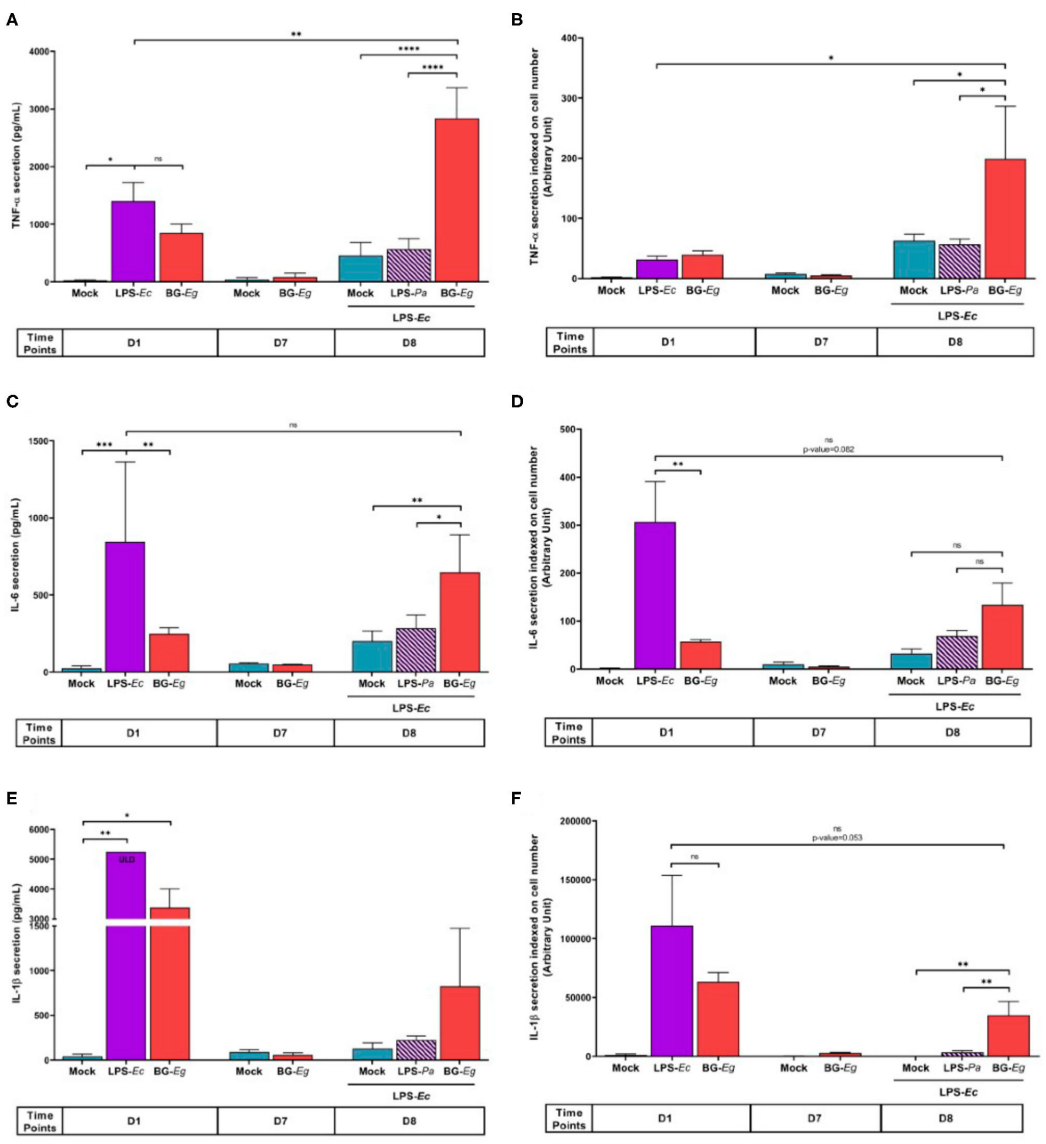
Trained immunity cytokinic signature throughout an immune training protocol. **(A)** TNF-α secretion (Means ± SD). **(B)** TNF-α secretion indexed on cell numbers. **(C)** IL-6 secretion (Means ± SD). **(D)** IL-6 secretion indexed on cell numbers. **(E)** IL-1β secretion (Means ± SD). **(F)** IL-1β secretion indexed on cell numbers. ANOVA tests *p*-values * < 0.05, ** < 0.01, *** < 0.001, **** < 0.0001.

The first stimulation with LPS-*Ec* and BG-*Eg* significantly enhanced TNF-α secretion compared to the control group at D1 (Figure 2A). No statistically significant difference can be highlighted at D1, after the two primings, though LPS-*Ec* stimulation increased the mean secretion of TNF-α by a 1.65 factor compared to BG-*Eg* stimulation. After the resting period on D7, all conditions were back to basal levels, confirming the transitional effect of the priming with an implementation of a latent state of the cells. On day 8, after the immune challenge, BG-*Eg*-primed macrophages exhibited a drastic increase in TNF-α secretion significantly different from both the control group (Mock/LPS-*Ec*) on the same day and LPS-primed macrophages (*p* < 0.0001 for both). This response to LPS-*Ec* challenge after a full immune training with BG-*Eg* was also enhanced compared to the first stimulation on fresh macrophages at day 1 (*p* < 0.01). When indexed on the number of cells (Figure 2B), this global effect of the immune training with BG-*Eg* was confirmed and showed that priming with BG-*Eg* enhanced TNF-α secretion in response to a danger signal.

IL-6 secretion throughout the immune training displayed a similar profile to the one observed with TNF-α (Figure 2C). On day 1, the first stimulation with BG-*Eg* was significantly lower than the one obtained with LPS-*Ec* stimulation (*p* < 0.01). The same return to basal secretion was observed on day 7, after the resting period for both conditions. After completion of the protocol, on day 8, unprimed cells re-stimulated with LPS-*Ec* (Mock/LPS) displayed a mean of IL-6 release significantly lower than the secretion by BG-*Eg*-trained macrophages (*p* < 0.01). Still, full training with BG-*Eg* failed to enhance the IL-6 secretion level compared to the first stimulation performed with LPS-*Ec* on day 1. IL-6 secretion indexed on cell number did not show an elevated response to LPS-*Ec* exposure whether it was compared to the Mock/LPS condition (day 8) or to the naïve cells exposed to LPS (day 1) (Figure 2D).

Priming with LPS-*Ec* induced a high IL-1β secretion that reached the upper limit of detection (ULD) of the ELISA test (5246.09 pg/mL) (Figure 2E). BG-*Eg* stimulation also induced a high IL-1β secretion lower than the one observed after LPS-*Ec* stimulation yet with no statistical significance. The resting period abolished IL-1β secretion, control cells and BG-*Eg*-primed cells recovered basal levels of secretion. At the end of the protocol, BG-*Eg*-primed macrophages exhibited a higher IL-1β secretion upon LPS-*Ec* re-stimulation compared to mock condition or to LPS-*Pa*-primed macrophages. No significant differences between the three conditions could be highlighted. Response to LPS-*Ec* exposure after a full immune training with BG-*Eg* did not increase in absolute number compared to the first exposure on day 2. Indexing on cell number did not change the general profile of IL-1β secretion throughout the protocol (Figure 2F). The first stimulation with BG-*Eg* exhibited a high index of IL-1β secretion which is not seen for the other cytokine, pointing in the direction of a specific role of IL-1β early secretion upon BG-Eg priming further discussed in this article.

The overall signature of IL-6, TNF-α, and IL-1β secretions after a full immune training with BG-*Eg* clearly showed an increased pro-inflammatory response to LPS exposure compared to non-primed cells.

**Figure 3:**
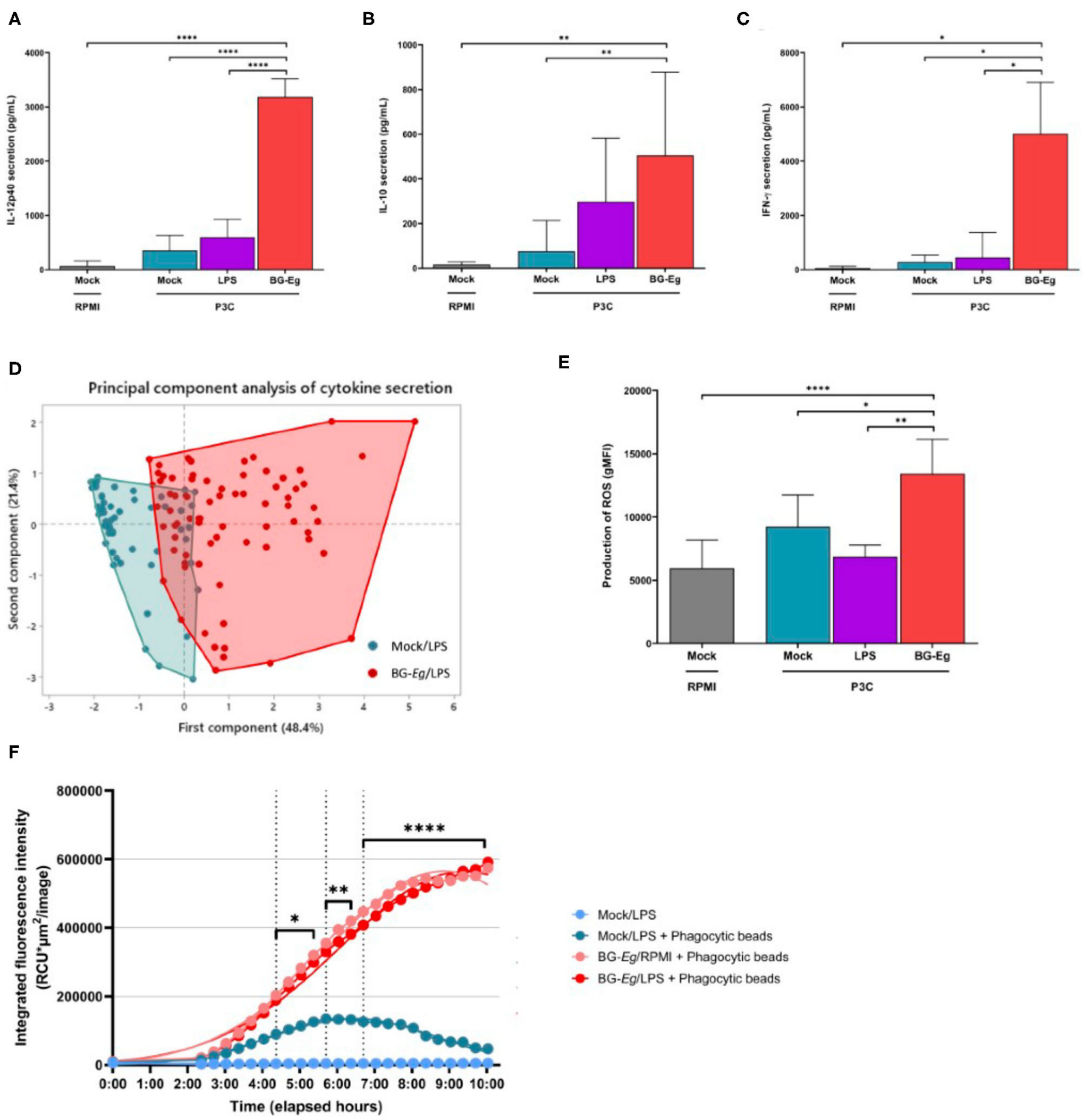
Pro-inflammatory and anti-microbial responses of canine macrophages after a full immune training. **(A)** IL12p40 secretion (Means ± SD). **(B)** IL-10 secretion (Means ± SD). **(C)** IFN-γ secretion (Means ± SD). **(D)** Principal component analysis of IL-6, IL-10, TNF-α and IFN-γ, secretion after a full immune training. **(E)** ROS production (geometric mean of fluorescence intensity ± SD). **(F)** Phagocytic activity (Total intensity of fluorescence of *E. coli* beads integrated per number of red-fluorescing cells). ANOVA tests *p*-values * < 0.05, ** < 0.01, *** < 0.001, **** < 0.0001.

We then analyzed the final secretion of IL-12, IL-10, and IFN-γ at the end of a full immune training with BG-*Eg*. The subunit IL-12p40 was measured in response to P3C challenge for three different conditions of priming: control with no priming (mock), LPS-primed and BG-*Eg*-primed macrophages (Figure 3A). BG-*Eg* priming significantly enhanced IL-12p40 secretion upon P3C stimulation compared to any other condition (*p* < 0.0001) confirming the global pro-inflammatory picture painted by the trained immunity-associated cytokinic signature. IL-10 secretion was consistently increased by BG-*Eg* priming, yet in a lesser extent than the other cytokines, with no statistical difference between LPS-primed macrophages or naïve macrophages (Figure 3B). We measured IFN-γ production by macrophages upon P3C exposure (Figure 3C). IFN-γ was found increased especially by macrophages previously primed with BG-*Eg*, following IL-12p40 pattern.

This first step of β-glucans screening enabled to characterize the first elementary signature of trained immunity by the enhanced secretion of pro-inflammatory cytokines illustrated by TNF-α. We, then, analyzed the full set of cytokines composed of IL-6, IFN-γ, IL-12p40, IL-10, and TNF-α in response to a full immune training. The complete profiles of cytokine secretion upon P3C re-stimulation for BG-*Eg* priming vs. mock condition are shown in the Figure 3D. This first component (48.4%) is mostly correlated with secretion of IL-6, IL-12p40 and TNF-α whereas the second one (21.4%) correlates with IL-10 secretion. IFN-γ release participates to both component in comparable proportion. Priming with the β-glucan from *E. gracilis* drives a progressive increase in the first component that shifts the repartition on the x axis of the graphical representation.

Based on the profiles aforementioned, the general trend induced by BG-Eg priming confirmed a heightened secretion of cytokines associated to Th1 and regulatory patterns. These results obtained with canine macrophages are aligned with the description of trained immunity in other species.

### Training With β-Glucan Increases Reactive Oxygen Species (ROS) Production and Phagocytic Activity of Macrophages

Pro-inflammatory profile of activated macrophages is often related to anti-microbial purposes. We investigated the activation status of macrophages upon full immune training by measuring the intracellular levels of reactive oxygen species (ROS) and phagocytic activity of canine macrophages.

Intracellular ROS production was measured by flow cytometry and results are shown in geometric means of fluorescence intensity (gMFI) (Figure 3E). Immune training with BG-*Eg* increased the intracellular level of ROS by 1.46 compared to mock condition and by 1.95 compared to LPS-*Ec*-primed condition. These results are statistically significant with *p* < 0.05 and <0.001, respectively. ROS production is thus consistent with the pro-inflammatory cytokine secretion observed upon full immune training with BG-*Eg* described in the upper section.

The phagocytic activity of macrophages was assessed in real-time using the Incucyte™ device coupled with fluorescent phagocytic beads of *E. coli*. After a full training protocol, cells were put in contact with *E. coli* particles which are attached to beads fluorescing upon phagocytosis and acidification of the phago-lysosomes. The red-fluorescing cells were counted and their mean intensity of fluorescence integrated per image. Mock/LPS condition with no priming showed peak of phagocytosis at 6 h after the exposure to the phagocytic beads. BG-*Eg*-primed macrophages were put in contact with phagocytic particles with or without prior LPS exposure (Figure 3F). Both conditions are overlaid with a greater integrated intensity of red fluorescent cells compared to mock condition, with significant statistical differences depending on the timing with *p*-values varying from <0.01 to <0.0001. The kinetics of phagocytosis also had a different profile upon BG-*Eg* training as the maximum of phagocytosis was not reached at the end at the recorded time.

Altogether, increase in ROS production and enhanced phagocytic capacity demonstrated a potent role of BG-*Eg*-primed canine macrophages in fighting against microbial assaults *in vitro*.

### Epigenetic and Metabolic Changes Regulate β-glucan Training of Canine Macrophages

**Figure 4:**
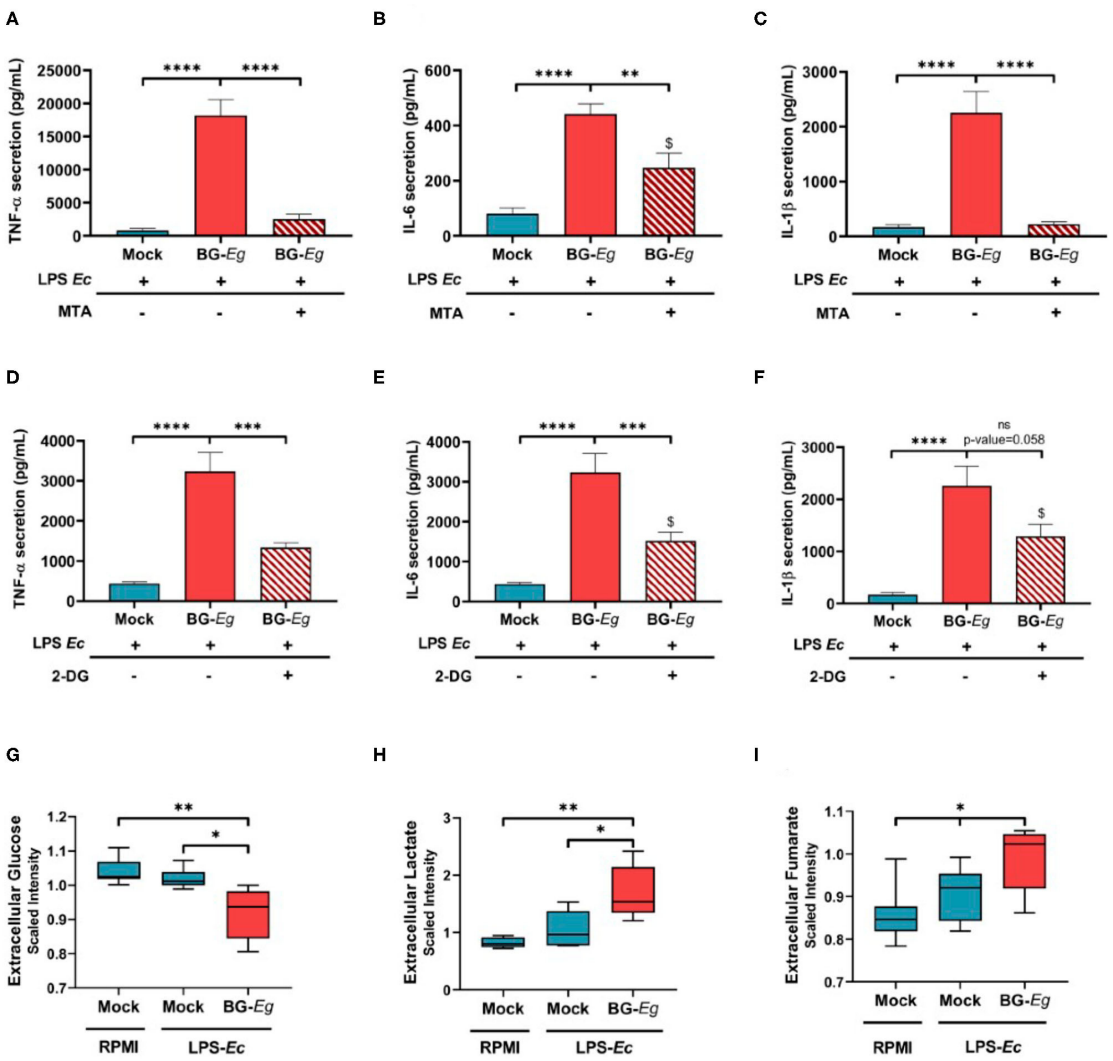
Epigenetic and metabolic features regulate trained immunity implementation in canine macrophages. **(A–C)** Inhibition of histone methylation by MTA abrogates the enhanced cytokine production (Means ± SD). **(D–F)** Inhibition of glycolysis by 2-DG abrogates the enhanced cytokine production (Means ± SD). **(G–I)** Glycolytic flux is increased by full immune training (Means of scaled intensity ± SD). ANOVA tests *p*-values * < 0.05, ** < 0.01, *** < 0.001, **** < 0.0001.

To show the implication of epigenetic mechanisms in BG-*Eg*-training of canine macrophages, we used inhibitors of these specific cell functions. First, by using 5′-deoxy-5′-methylthioadenosine (MTA), we inhibited post-translational methylation of histones. As previously shown, immune training with BG-*Eg* increased the secretion of TNFα, IL-6, and IL-1β compared to mock conditions. This effect is almost completely abrogated upon use of MTA prior to BG-*Eg* incubation, with a significant decrease of TNF-α secretion (Figure 4A) and IL-1β secretion (Figure 4C; *p* < 0.0001 for both results). The inhibition of methyltransferases by MTA also reduced significantly the secretion of IL-6 (Figure 4B). Glycolysis inhibition by 2-deoxy-D-glucose (2-DG), during immune training with BG-*Eg* resulted in similar profiles, significantly decreasing TNF-α (Figure 4D) and IL-6 release (Figure 4E) (both *p* < 0.001). IL-1β secretion was also diminished by the use of 2-DG yet not significantly compared to Mock/LPS condition (Figure 4F).

Altogether, these results indicated the imperative need for histone methylation and enhanced glycolytic rate to implement the trained phenotype in canine macrophages.

To further investigate the role of metabolic changes in the immune training, we assessed the glycolytic function and its downstream metabolites after immune challenge. Firstly, we measured glucose and lactate concentrations in cell culture supernatants to look at the entryway and the end product of glycolysis. Glucose concentrations in supernatant is significantly lower in cells that underwent a full immune training compared to both mock conditions with or without LPS re-stimulation (Figure 4G; *p* < 0.05). At the same time, lactate production is significantly higher in trained macrophages than in unprimed conditions (Figure 4H; *p* < 0.05). Fumarate, an endpoint metabolite in the urea cycle was also found increased upon full BG-*Eg*/LPS training compared to control condition (Figure 4I; *p* < 0.05). Altogether these results show the implication of metabolic changes in trained immunity, with a particular importance of glycolysis enhancement, even in a context of oxygen abundance, a process named the Warburg effect.

## Discussion

The presented work demonstrated for the first time that canine macrophages are able to undergo immune training in response to β-glucan stimulation. We were able to show that the three pro-inflammatory cytokines (TNF-α, IL-6, and IL-1β), hallmark of the trained immunity signature are significantly up-regulated upon training with β-glucan. These results are the first demonstration of such effect of trained immunity in dogs.

The role of IL-1β seemed of particular importance in this context. Indeed, it has been documented that IL-1β could act in an autocrine and paracrine manner to implement and further reinforce the trained immunity phenotype (30). In this study, we showed that IL-1β secretion in response to β-glucan stimulation on naïve macrophages was greatly enhanced. Although LPS induced a higher secretion of IL-1β in naïve macrophages, there was no statistical difference between the secretion levels in response to LPS and β-glucans. Interestingly, it is the only cytokine for which a priming stimulation with β-glucan was higher than the secretion observed at the end of the immune training. This could point out a specific role for this cytokine as trained immunity inducer. Indexing the secretion on cell number confirmed these observations. After the resting period, cells exhibited very low cytokine secretion indicating an abrogation of the immuno-stimulation, this returned to baseline production securing the lack of constitutive activation. This resting period was crucial for the distinction between immuno-stimulation and immune training. On the one-hand, it allowed the cells to undergo the intracellular modifications characteristic of trained immunity and on the other hand, it provided the necessary time to dissipate the effects of the primary immuno-stimulation.

Treatment with P3C was used as a secondary immune challenge on the cells previously trained with BG-*Eg, LPS* or not trained (mock condition). We measured additional cytokines to further decipher the immune activation by β-glucan and LPS. IFN- γ and IL-12 are cytokines greatly implicated in immune response to pathogens, with a specific macrophage stimulating role of IFN-γ that could further activate the innate immune responses. The implication of such cytokines activation would need to be further investigated in the context of specific immune response to vaccination.

To further demonstrate the ability to fight pathogens, we investigated anti-microbial responses through the analysis of ROS production and phagocytic activity. Both parameters were found increased upon β-glucan training with statistical significance. Anti-microbial responses during an infection plays a pivotal role in controlling pathogens before the activation of the adaptive immunity and the cellular cooperation that comes with it. Macrophages are able to produce a large amount of anti-microbial compounds, such as enzymes, ROS, prostaglandins that will act by directly targeting intruding organisms. Trained immunity has been shown to enhance the ability of macrophage to secrete anti-microbial compounds, mainly by the metabolic reprogramming associated with it and in particular modifications of mitochondrial activities (31, 32).

The use of inhibitors of histone methylation during the training of the cells completely abolished the effect on cytokine secretion. It has been shown in human and mice that upon a first stimulation or infection, naive cells will present a priming state that will activate post-translational modifications on histone proteins (33). These modifications establish the ability of cells to launch a faster and higher inflammatory response upon a second stimulation. Histone methyltransferases (HMT) responsible for histone methylation require S-Adenosyl methionine (SAM) as both a cofactor and a methyl donor group. Various metabolites, such as fumarate and short-chain fatty acid in general, have been reported to function as cofactors for epigenetic enzymes, which in turn induce chromatin and DNA modification which eventually modulate gene expression (34). Studies also employed epigenetics to elucidate the role of immuno-metabolism in trained immunity and showed that H3K4me3, marker of active euchromatin, is more present on promoters of glycolysis genes (18, 21). A similar pattern was seen on the IL-6 and TNF-α promoters. Notably, the marks on these pro-inflammatory cytokine genes returned to baseline when inhibitors of glycolysis were used. Therefore, while immune training is required to alter cell metabolism, these metabolic changes in turn seem to be necessary to maintain a trained phenotype (16, 35).

Metabolic modifications of trained cells were then investigated. Glycolytic rate was determined by assessing the glucose consumption from cell culture supernatant and lactate production (the end-product of glycolysis) was measured as a function of the extracellular acidification rate. Pro-inflammatory macrophages have a tendency toward increased glycolysis and lactate production, while anti-inflammatory macrophages appear to depend more on oxidative phosphorylation and lipid metabolism (36). Similarly, trained macrophages shift to a pro-inflammatory phenotype. Glucose consumption increased upon secondary stimulation of these cells, mirrored by a proportional increase in lactate.

Immune training and the metabolic changes associated with it seem to implement a sort of regulatory loop, thereby enhancing each other (22). Indeed, glutamine metabolism, already described as a key mechanism of macrophage polarization, also regulates trained immunity through enhanced fumarate production further signaling through Akt/mTOR and regulating chromatin methylation (37). The increased glycolysis observed in trained immunity will eventually head toward an accumulation of citric acid cycle substrates such as fumarate (16). Fumarate has been shown to stabilize HIF-1α (hypoxia-inducible factor 1α), a transcription factor of distinctive importance in regulating glycolytic genes and IL-1β production (21). Both fumarate and lactate have been described in the inhibition of histone demethylases and deacetylase, respectively (20, 38). The metabolism shift implemented with trained immunity lead to an enhanced glycolytic flux generating more metabolites susceptible to be used as cofactors to chromatin-modifying enzymes. Yet, the timing and causality of these observations remain unclear and need to be further documented to better understand immuno-metabolism in the context of trained innate immunity and implement it in clinical settings.

Amongst the β-glucans tested in this study, the one extracted from *E. gracilis* (BG-Eg) displays the highest production of pro-inflammatory cytokines, statistically different from every other β-glucans. Interestingly, Curdlan shares the same structure, also a linear β-(1, 3) polymer of D-glucose and show the second best signature of trained immunity. Zymosan, which possesses β-(1, 6) ramifications on a linear β-(1, 3) backbone (39), show less intensity in the increase of pro-inflammatory cytokines secretion. Laminarin and whole glucan particle both constituted of branched polymer failed to implement trained immunity signature as well as Dextran, an α-(1, 6)-D-glucose with α(1, 3)-linkages. These results can be linked to the analysis of canine Dectin-1 conformation, the cell-surface receptor of β-glucan. These differences in subsequent biological activities of β-glucans could be attributed to dissimilarities in their capacity of binding to the Dectin-1 given its conformation (40). As mentioned earlier, Dectin-1 structures are highly conserved among studied species. Yet, notable differences in the groove of the binding site, reduce accessibility in the canine protein (41). These changes in the groove shape tend to change the interaction site accessibility, therefore only smaller compounds could interact with the bottom of the active site compared to Dectin-1 from mice or humans. These 3-D structures differences could explain the lack of efficiency of ramified β-glucan to induce a biochemical response in dogs as found in the presented model with Laminarin, Dextran or WGP. These findings are consistent with published data in which human and murine immune training can be accomplished with branched β-glucans (42) whereas linear ones seem to be the most effective in our dog model. Few data exists on the length of β-glucans that bind to their receptor Dectin-1 though very high molecular weight and thus high number of residuals seem to compromise their binding (14, 43).

The general implications of branching, molecular weight and conformation of β-glucan is still unclear for the most part although linear chains or poorly branched molecules seem to display the highest biological activity throughout all models (40). The differences between species' isoforms of Dectin-1, the preferential receptor alone or associated to co-receptors for β-glucans, could partly explain this issue. Indeed, in this study, we show that conformational predictions are dissimilar between mice, human and dogs in terms of accessibility to the binding site. This reinforce the need to better characterize β-glucans structures and characteristics that would allow a standardization of nomenclature of these complex molecules.

Overall the enhancement of canine macrophages' immune responses upon β-glucan training are comparable to what has been described in other mammal species, mainly human and mice, but also in fish (44). This is the first demonstration of trained immunity in dogs and therefore helps to consolidate the implication of trained immunity in host defense mechanisms. The general features of metabolism and epigenetic regulation are also shared in-between species, yet, specific differences, such as receptor conformation, seem to play a role in the modulation of trained immunity throughout evolution. Trained immunity opens new scope of investigations from the newly theorized trained immunity-based vaccines to the long-lasting controversy of non-specific effects of vaccines (23, 45).

## Data Availability Statement

All datasets presented in this study are included in the article/supplementary material.

## Ethics Statement

The animal study was reviewed and approved by The Boehringer Ingelheim Ethical Committee (registered under n° 13 at the French Ministère de l'Education Nationale, de l'Enseignement Supérieur de la Recherche) has reviewed all animal experiments conducted for this study before conducting the study. Approval confirms that all experiments conform to the relevant regulatory standards defined by the European and French Laws (directive EU2010/63 and Decret n°2013-118).

## Author Contributions

SP, MP, ML, and LC performed the research. SP, KD, and LF designed the research and analyzed data. TS and RS analyzed the data. CS-C performed the modeling of Dectin-1 structures. SP, KD, and CS-C wrote the paper. All authors contributed to the manuscript review. All authors contributed to the article and approved the submitted version.

## Conflict of Interest

TS and RS were employed by the company Lifebit Biotech Ltd. SP, LC, MP, CS-C, HP and KD were employed by Boehringer Ingelheim Animal Health. The remaining authors declare that the research was conducted in the absence of any commercial or financial relationships that could be construed as a potential conflict of interest.
